# Ethical considerations and the social context of HIV phylogenetic research in uMkhanyakude district, KwaZulu-Natal, South Africa

**DOI:** 10.4102/sajhivmed.v27i1.1826

**Published:** 2026-09-23

**Authors:** Rujeko Samanthia Chimukuche, Thembelihle Zuma, Qinisile Shandu, Manono Luthuli, Dumisani Gumede, Janet Seeley

**Affiliations:** 1 Social Sciences Core, Africa Health Research Institute, Durban, South Africa; 2 Division of Infection and Immunity, University College London, London, United Kingdom; 3 School of Nursing and Public Health, College of Health Sciences, University of KwaZulu-Natal, Durban, South Africa; 4 London School of Hygiene and Tropical Medicine, London, United Kingdom

**Keywords:** ethics, HIV, phylogenetics, social contexts, social risks and harms

## Abstract

**Background:**

HIV phylogenetic analyses provide insights into genetic relatedness and transmission patterns but cannot directly identify specific transmission events or the direction of transmission. However, if not well understood and handled carefully, phylogenetic results have the potential to harm individuals and groups.

**Objectives:**

The study aimed to explore the ethical considerations and the social context of HIV phylogenetic research.

**Method:**

A qualitative methods study on phylogenetics was embedded within a clinical trial on community-based biosocial HIV prevention and sexual reproductive health for youth in rural KwaZulu-Natal, South Africa. Data collection was conducted over an 8-week period using in-depth interviews. Data were audio-recorded and translated verbatim and transcribed into English in anonymised format. Data were later analysed thematically. Four themes were identified: perceived benefits of phylogenetics, informed consent, participant privacy and data security and social risks.

**Results:**

Participants acknowledged the benefits of using phylogenetics in HIV research highlighting the importance of identifying transmission pathways in high prevalence settings. However, consideration should be given to community members with informed consent required.

**Conclusion:**

Ethical care is necessary in HIV phylogenetic research. Strong ethical and rights-based frameworks that facilitate prevention strategies while respecting the rights and interests of individuals and minimising any risks are required.

**What this study adds:** This study offers insights into the ethical use of HIV phylogenetics in public health, epidemiological studies, clinical trials, and individual clinical management. Findings offer suggestions on how to minimise risks for analyses that generate sensitive data.

## Introduction

Phylogenetic analysis determines if pathogens found in two individuals are closely related to each other or not, providing insights into how pathogens travel between human and animal hosts and how they change in the process.^1^ Phylogenetic analysis was used during the COVID-19 pandemic to investigate the transmissibility and patterns of movement of severe acute respiratory syndrome coronavirus 2 (SARS-CoV-2) infections with a view to addressing the spread of the virus.^2,3^ Similarly, the spread of HIV, which came to prominence as a global pandemic in the 1980s, has also been traced using phylogenetics, providing a valuable tool for understanding transmission dynamics and tailoring interventions.^4,5^ HIV phylogenetics can be used to understand the HIV epidemic and the risks of transmission in different settings, focusing interventions where needed.^6^ HIV phylogenetics can reveal how HIV spreads in a population.

Despite the potential benefits, the use of phylogenetics raises important ethical concerns,^7^ given the long history of stigmatising particular groups and occupations for the transmission of diseases.^8,9,10,11,12^ Indeed, during COVID-19, as the spread of the virus was tracked, blame for the spread was initially attributed to particular nationalities and groups, such as international travellers,^13,14,15^ and to particular forms of behaviour^16^ as well as to the places where a variant was first identified.^17^

The main ethical challenge in HIV phylogenetics is balancing the need to understand transmission dynamics with the potential risk of unintentionally compromising personal privacy through the use of sensitive data.^18,19,20,21^ The potential harms that could arise from disclosing results, particularly any suggestion of direction of infection, could be personally harmful to the research participants because of social stigma and criminalisation surrounding HIV,^22,23^ leading some researchers to choose not to share their findings because of the potential harm that could be caused to certain groups in a community and to avoid ‘eroding the trust between phylogenetic researchers and communities of people living with HIV’.^24^ Tordoff et al.^24^ demonstrate how concerns about stigma and community trust can influence decisions about the dissemination of phylogenetic research findings. Following community engagement, the researchers chose not to publish their original study because they concluded that the potential harms, such as reinforcing stigma and undermining trust between researchers and communities of people living with HIV, outweighed the anticipated public health benefits.

In South Africa, the law governing genomic research is contained in several sections of the legislation,^25^ including the *Constitution of the Republic of South Africa*,^26^ which grants a right to privacy, the *National Health Act, 61 of 2003*,^27^ and the *Protection of Personal Information Act (POPIA), 4 of 2013*, enacted in 2019.^28^ Importantly, South African legislation protects against discrimination and stigmatisation especially regarding one’s genetic material.^25,27,29,30^ These legal frameworks help researchers to incorporate confidentiality, ethical approvals and clear consent processes into study designs. In terms of data use, legislation such as *POPIA* restricts how genomic information can be stored and shared, making secondary use and open data sharing regulated because of risks of re-identification. These regulations create strong safeguards for participants and encourage governance of phylogenetic data.

In this analysis we explore the ethics of HIV phylogenetic research, the potential benefits and harms relating to its use, and the social risks that arise when conducting this research in rural settings in KwaZulu-Natal (KZN), South Africa. We further draw lessons for the ethical conduct of phylogenetic methods to protect individuals and groups at risk of stigma and blame.

## Research methods and design

### Data collection, sampling, and recruitment

Participants were identified using a purposive sampling strategy based on three pre-determined categories: healthcare workers, community members, and Africa Health Research Institute (AHRI) community advisory board (CAB) members. Participation in the qualitative study was not limited to trial participants (details of the trial are given below). Community members were recruited from the same study communities, and while some adolescents and young people may have participated in the parent trial, trial participation was neither assessed nor required for inclusion in the qualitative study. The qualitative sample was purposively selected to capture a range of perspectives from community members, CAB members, and healthcare workers regarding the ethical and social implications of HIV phylogenetic research. These categories enabled the researchers to extract a variety of perceptions and experiences regarding ethical considerations of phylogenetics research in the study area. Community advisory board members were selected randomly because they have oversight of the entire study area and we recruited them for interviews until data saturation was reached. Community members were purposively recruited from semi-urban and deep rural communities to contrast findings among individuals from different backgrounds.

Healthcare workers were purposively selected to have a balanced mix of gender and nursing qualifications (enrolled nurses and registered nurses). The Africa Health Research Institute (AHRI) has a memorandum of understanding with the Department of Health which stipulates that nurses should have an equal opportunity to participate in AHRI research every year. The AHRI nursing manager identified nurses who had not had an opportunity to participate in research during the year 2022 and provided the research team with their contact information. The nurses were contacted and invited for interviews at their convenience.

A presentation about the study was conducted by study leads to the CAB, and members of the CAB were invited to volunteer to participate. Before the interviews, participants were provided with an overview of the trial on which the scenarios were based to ensure a shared understanding of the study context in which they were invited to volunteer to participate. As they volunteered, we sought to balance the age and gender to get an even mix of younger and older male and female participants. For community members, we purposively sampled to identify a balance of older and younger male and female participants from age 16 and above within two rural communities and two peri-urban communities.

Participants were asked about their understanding of phylogenetics, including how it can be used to understand HIV evolution, responses to treatment and prevention interventions, and transmission within and across communities. Discussions were facilitated using three hypothetical scenarios illustrating potential applications of whole HIV genome sequencing among young people. The first scenario explored the retrospective and anonymous use of whole HIV genome sequencing to assess whether individuals exposed to the intervention arm of a trial were less likely to contribute to HIV transmission networks. The second scenario considered the real-time, anonymous use of whole genome sequencing to identify populations that could benefit from pre-exposure prophylaxis (PrEP). The third scenario examined the use of whole genome sequencing to understand HIV viral evolution under drug pressure associated with real-world use of PrEP and antiretroviral therapy (ART). Participants were also asked about their perceptions of the ethical and social implications associated with these potential applications.

The multilevel HIV prevention team in northern KZN undertook a stepped-wedge clinical trial to evaluate community-based biosocial HIV prevention and sexual reproductive health for youth in rural KZN, South Africa.^31^ A phylogenetics qualitative, using in-depth interviews for data collection study was an embedded sub-study within the main trial.

Data collection took place over an 8-week period from 01 July 2022 to 31 August 2022. We first investigated how whole genome sequencing among young people could be used, both anonymously and retrospectively, to understand HIV transmission networks. Second, we investigated how phylogenetic data could be used to identify young people who may benefit from PrEP in real time and anonymously. Third, we looked at how phylogenetic data could be used to understand the virus evolution under drug pressure from real-world use of PrEP or ART. Discussions included participants’ perceptions around legal, social, and ethical issues associated with this approach.

A semi-structured topic guide was used for the interviews. Social science researchers conducted interviews in the local language (isiZulu) and translated into English. Translation accuracy was then ensured through a two-step quality control process, in which two senior field staff independently reviewed the translated transcripts for consistency, meaning and linguistic accuracy. Any discrepancies were discussed and resolved through consensus to ensure fidelity to the original data. The interview guide was developed by the multidisciplinary study team based on the study objectives and existing literature on the ethical and social implications of HIV phylogenetics. Prior to data collection, the guide and hypothetical scenarios were piloted with mock participants who were employees of the institution and residents of the study communities. The pilot was used to assess the clarity, appropriateness, and cultural relevance of the questions, evaluate participants’ understanding of phylogenetics concepts and terminology, determine the flow and sequencing of the interview, estimate interview duration, and identify any potentially confusing or sensitive content. Feedback from the pilot informed revisions to the wording of questions, explanations of phylogenetics, and the presentation of the hypothetical scenarios before data collection commenced. Each interview lasted between 45 min and 60 min. Informed consent was sought from each participant after the purpose of the study was explained. Participants were also given time to review information on the study prior to giving their written informed consent to be engaged in the interview process.

Data were digitally recorded, transcribed and translated into English and stored on a password-protected data server. Interview summaries were written by the social science researchers immediately after each interview. These summaries provided an overview of the conduct of the interview and the main points raised in order to complement the transcription after processing.

### Data analysis and interpretation

Data analysis was done manually, led by the first author, with preliminary coding done by Qinisile Shandu and Manono Luthuli; reviewing of the analysis was conducted by Thembelihle Zuma. The coding framework was initially informed by the interview guide and then expanded and refined based on the team’s review of the transcripts to capture emerging codes. The main codes were reviewed, discussed, and agreed by consensus for validity, and similar codes were merged to avoid duplication.

While the analysis was primarily deductive, where relevant, additional codes were generated inductively to capture insights. Manual coding was chosen because it enabled close analysis with the data and facilitated interpretation of participants’ perspectives, which was particularly important given the exploratory nature of the study. We prepared detailed analytical memos which documented the coding process and how we developed the themes. To increase reliability and validity, the main codes were reviewed and discussed. Related deductive and inductive codes were compared across participant groups and organised into broader analytic categories.

Major themes were identified. A coding framework was developed in Microsoft Excel, on which the coded data were manually inserted by pasting information and illustrative quotes from the interviews against the matching themes. All data were anonymised, and participants were identified only by their sex, age, and interview round in this study. When interpreting the findings, team members who collected the data and were familiar with the setting helped provide context and deeper understanding of participants’ responses. At the same time, they did not assume meaning beyond that which the participants shared. To enhance reflexivity, the research team held regular debriefing meetings throughout data collection and analysis to reflect on how their backgrounds, prior community engagement, and assumptions might influence data collection and interpretation. Reflexive field notes were taken, discussed during team meetings, and incorporated into the analytic process to support transparent and contextually grounded interpretation of the findings.

### Ethical considerations

The study was approved by the University of KwaZulu-Natal Biomedical Research Ethics Committee, approval number BREC/00003735/2021. Written informed consent was obtained from all individual participants involved in the study.

## Results

Fifty-five in-depth interviews (Table 1) were conducted with consenting individuals including 40 purposively recruited community members, 5 CAB members, and 10 healthcare workers. The findings were grouped under four main themes: perceived benefits of phylogenetics, informed consent, participant privacy, and data security and social risks.

**TABLE 1:** Study participants (*N* = 55), KwaZulu-Natal, 2022.

Category of participants	Age range (years)	Location
Community members (*n* = 40)	16–65	Machibini (*n* = 10), Ogengele (*n* = 10), KwaMsane (*n* = 10), KwaMsane Reserve (*n* = 10)
Healthcare workers (*n* = 10)	28–55	Somkhele Clinic (*n* = 2), Mtuba Clinic (*n* = 2), Machibini Clinic (*n* = 2), Mpukunyoni Clinic (*n* = 2), KwaMsane Clinic (*n* = 2)
Community Advisory Board members (*n* = 5)	27–61	Ogengele (*n* = 1), KwaMsane Reserve (*n* = 1), Baswazini (*n* = 1), Myeki (*n* = 1), Ophaphasi (*n* = 1)

### Perceived benefits of phylogenetics

Participants shared that phylogenetics could help couples, families, and communities to understand transmission trends and different HIV strains. The participants, emphasised their perception on the disclosure of results in controlling the spread of the virus:

‘[*I*]t can help in knowing that the virus is spreading more at [*location blinded*] because if you combine them, you will not be able to see where the virus is spreading more, it might seem easy when you look at it like that. So that you will be able to fight that problem you have, to know where and who has it so that you will finish this [*pandemic*].’ (P4, male, community member)

Participants further highlighted that by conducting phylogenetics retrospectively, there will be a delay in promoting appropriate behaviour change and necessary biomedical interventions. However, the information was still perceived to be helpful:

‘Yes, it will be very much helpful. It will help us by discovering how many people are infected by that time and how many should start the treatment.’ (HCW 3, female, enrolled nurse)

Several healthcare workers perceived that phylogenetics would be useful in real time by providing crucial information about HIV prevalence, severity, transmission, possible treatment, and understanding treatment efficacy. These perceptions were influenced by the experiences observed during the COVID-19 pandemic.

Other benefits shared included the possibility of phylogenetics assisting with identifying drug-resistant and other characteristics of HIV before treatment is initiated and improving the identification and tracking of sexual partners who could be at risk:

‘In my opinion it would be best if everyone who participates in the phylogenetic research receives their results. If we make it optional and we find someone to have a lethal fast spreading variant, it will be dangerous because with communicable diseases, you often first infect those closest to you like your family.’ (HCW 2, male, professional nurse)

Such concerns raise anxieties around involuntary HIV disclosure that are rooted not only in HIV phylogenetics, but also in historical experiences of stigma, blame, and social risks associated with being ‘outed’.

### Informed consent

Community advisory board members highlighted that identifying key transmission groups is important, as long as the information given during the consent process is comprehensive and understood by participants. This was seen to be particularly important in the context of sharing results from the study:

‘I think the part of telling should be the agreement between the both of you before the research commences if he will be willing to know his results, if he wishes to know them how does he prefer to know them, he should know it from his partners or he should be the only one knowing.’ (P4, male, CAB member)

Some CAB members and other community members stressed the importance of thorough community engagement about the use of phylogenetics in an ongoing process while the method was being used, not only when seeking consent to take part in a phylogenetic study.

Participants also showed their awareness of their right to agree or decline participation, by suggesting that informed consent may be understood more as an agreement:

‘It can be good because you know your intentions for what you want, I think it is alright for you to get what you want. Because research came to us, they want the information if I agree with my blood they write and store that information, this is what we found.’ (P4, male, community member)

This reflects that the informed consent process is highly important. While participants can show their autonomy in decision-making, they have a choice whether to agree or not.

### Participant privacy and data security

Participants stressed the importance of highlighting confidentiality in the informed consent process:

‘I think the generation we are in would not [*agree*] to be exposed and their names revealed because I do not think there is anyone who will agree to [*have*] their blood taken and put out there for the public to see who it is. No one can agree to that … it is personal, you cannot do that even if we agree you cannot show my name. So, I think it will be difficult to convince them, but maybe if you explain to them clearly that we will not reveal the name of the person, it is just that we want to make samples so that we can see some of you and be inspired.’ (P21, male, community member)

A number of participants stressed concerns about the possible loss of anonymity and privacy in phylogenetic research if confidentiality was not maintained. Community members feared that despite assurances of confidentiality, the linkage of samples to personal data could expose individuals’ biodata and personal details:

‘The danger can be what, it can be maybe you can walk in the dark without knowing whose blood is it or even if you will not go around telling people but that bottle will be named and given all necessary information about the participant.’ (P4, male, community member)

Participants also highlighted their uncertainty and mistrust about what is being done with biological samples (e.g. blood). There was a perception that researchers may unintentionally disclose whose sample it is, or that the sample might be mishandled or used in a way that those providing the sample had not agreed to.

While some CAB members and members of the community had reservations about the sharing of results, the healthcare workers expressed that there was an ethical responsibility to inform people about their results after blood has been collected:

‘I think if you are going to take someone’s blood after you have told them about all the potential harms and benefits of a study, that person deserves to know the results that emerged from their study.’ (HCW 2, male, professional nurse)

### Social risks

There were fears that phylogenetic results have the potential to harm individuals and groups. Our analysis revealed that phylogenetic studies could be misused to increase self-harm and intentional spread of the virus. A CAB member highlighted that self-harm could arise from disclosure of results:

‘Yes, they could kill themselves, because when a person thinks they will wonder what are they living for because they will die at the end and opt for suicide. They can kill themselves indeed, “your results are back … do you see that you are the one who is spreading?”’ (P6, female, CAB member)

This participant highlights the stigma-related risks that are associated with the disclosure and interpretation of phylogenetic findings, especially if individuals perceive that they are being told that they are responsible for the transmission.

Community members highlighted the potential dangers that could arise from disclosing the results to participants:

‘People shouldn’t be told; no one would agree to sit down and let it go and not approach someone who infected them with a virus. We would die.’ (P25, female, community member)

Others emphasised that although phylogenetic research has mental health risks and harm associated with it, the benefits may outweigh these risks because the outcomes could be beneficial:

‘The risk will be if people come to know about my status. Maybe I might end up taking action, because people are now aware that I am the one who came with this thing [*virus*], I might end up dying … killed by the fact that people are aware that I am one who is killing them. Yes, it has risks, but I would like to know the outcome.’ (P26, female, community member)

The participants in this study considered that the use of HIV-related phylogenetic research posed a number of risks to people’s privacy and well-being. Even so, some, such as the participant above, thought that despite the psychological distress driven by fear of stigma and social exposure related to HIV status, the use of phylogenetic analysis could still be beneficial in addressing continued HIV acquisition in the study setting.

## Discussion

Our results show participant understanding of the benefits of phylogenetics in HIV studies and the disadvantages that could arise from the disclosure of results. While phylogenetic analysis offers valuable insights into transmission tracing, its application in rural African communities, particularly in settings that may have highly stigmatised populations, such as sex workers, can raise concerns around participant privacy, informed consent, confidentiality, and the potential for harm as shown in other studies.^21,23,32^ These studies have shown that the use of HIV phylogenetics should be understood within broader contexts of stigma and criminalisation.^21,23^ Further, phylogenetic methods can provide insights into transmission patterns; however, they have important limitations such as individual responsibility for transmission.^22^ Informed consent is required to ensure that participants have made a voluntary choice to be involved based on a good understanding of how data will be collected, stored, used for research, or shared with others. Other studies have highlighted these concerns specifically related to privacy, confidentiality, and HIV disclosure, with some participants choosing to withdraw from phylogenetic research.^33,34,35^

Phylogenetic data should be interpreted in social contexts to avoid the risk of reinforcing harmful narratives.^36^ Caution can be exercised over how findings are communicated, ensuring that the language used does not contribute to further stigma as illustrated in other studies.^21,36^ To exercise this caution, the literature suggests that providing clear and tailor-made explanations using native language and simplified visual storytelling can enhance understanding and thereby reduce any risk of harm resulting from misunderstanding or misinterpretation.^37^ Concerns were raised in other studies,^36^ that if care is not taken, community members may avoid positive health-seeking behaviours that could benefit them and the population out of fear of stigma or fear that their involvement alone could lead others to suspect they are living with HIV or involved in stigmatised behaviour.^36^ This anticipated stigma could deter engagement, even when ethical precautions are in place.^38^

In phylogenetic research, it is important to implement ethical precautions and psychosocial support. In this study, participants expressed conflict between perceived benefits and anticipated mental health risks that could arise when an individual is involved in the research. The results highlight the need for adherence to ethical best practice in the conduct of the research, the provision of mental health support and guidance for the conduct of HIV phylogenetics.^21^ Additionally, effective informed consent procedures, thorough anonymisation of data, are essential to protect participants from harm.^21,36,39^

In our study, participants feared that phylogenetic data could lead to exposure of transmission networks as shown in other ethics studies.^7,21^ This concern reflects more than uncertainty about the technology but is associated with fears of involuntary HIV disclosure and stigma.^40^ Acceptance of phylogenetic approaches depends not only on scientific understanding but also on trust and confidence that institutions will use data ethically.

Future phylogenetic trials involving real-time contact tracing would need to carefully address historical fears of involuntary disclosure. Participants may interpret such approaches as surveillance and exposure of transmission networks thereby increasing potential social harm. Health policies can address concerns surrounding phylogenetic trials and real-time contact tracing by establishing strong ethics on confidentiality and data protection.^41^ Regulatory frameworks should be put in place to ensure that phylogenetic data are used for research and public health purposes and not used for involuntary disclosure of participants’ HIV status.^33^ Policies should also require informed consent processes that explain clearly what phylogenetic analysis and confidentiality are.^20^ Community engagement can assist in building trust and addressing participant concerns.^42^

### Limitations

A limitation of this study is that participants’ perceptions of phylogenetics were explored based on their understanding of the method, which often differed from its actual scientific capabilities. Many participants perceived phylogenetics as a tool that could directly identify who infected whom or definitively establish transmission events. Researchers acknowledge that, while HIV phylogenetic analysis can identify genetic relationships among viral sequences and provide insights into transmission dynamics, it cannot on its own determine direct transmission events, the direction of transmission, or conclusively identify the source of an individual’s infection. These perceptions are nevertheless important, as they shape community attitudes toward the acceptability, risks, and potential consequences of HIV phylogenetic research.

## Conclusion

A careful balance is required between the benefits and risks associated with the use of phylogenetics in research. Ethical conduct requires minimising social harms, including stigma, discrimination and lack of confidentiality when interpreting and communicating the findings. Minimising social risks and stigma linked to phylogenetic research is essential with an understanding of the social contexts in which the research is conducted, to make sure that communication of findings does not cause harm to participants. Communities need to be meaningfully engaged to promote transparency, build trust, and guide the ethical use of phylogenetic data.
